# Pharyngeal Gonorrhoea in Women: An Important Reservoir for Increasing *Neisseria gonorrhoea* Prevalence in Urban Australian Heterosexuals?

**DOI:** 10.1155/2013/967471

**Published:** 2013-06-26

**Authors:** M. Josephine Lusk, Ruby N. N. Uddin, Monica M. Lahra, Frances L. Garden, Ratan L. Kundu, Pam Konecny

**Affiliations:** ^1^Short Street Sexual Health Centre, Department of Infectious Diseases, Immunology and Sexual Health, St. George Hospital, Kogarah, Sydney, NSW 2217, Australia; ^2^St. George Clinical School, Faculty of Medicine, University of New South Wales, Randwick, Sydney, NSW 2052, Australia; ^3^WHO Collaborating Centre for STD, Microbiology Department, South Eastern Area Laboratory Services, Prince of Wales Hospital, Randwick, Sydney, NSW 2031, Australia; ^4^Sydney School of Public Health, The University of Sydney, Sydney, NSW 2006, Australia

## Abstract

We aim to characterize sexual behavioral aspects of heterosexual *Neisseria gonorrhoea* (NG) acquisition in two Sexually Transmitted Diseases clinics in Sydney, Australia, in 2008–2012. Of 167 NG cases, 102 were heterosexually acquired with a trend of increasing NG prevalence in heterosexuals from 1.1% (95% CI 0.6–2.1) in 2008 to 3.0% (95% CI 2.0–4.0) in 2012 (*P* = 0.027). Of heterosexual male cases, unprotected fellatio was the likely sexual activity for NG acquisition in 21/69 (30.4%) and commercial sex work (CSW) contact the likely source in 28/69 (40.6%). NG prevalence overall in CSW (2.2%) was not significantly higher than in non-CSW (1.2%) (*P* = 0.15), but in 2012 there was a significant increase in NG prevalence in CSW (8.6%) compared to non-CSW (1.6%) (*P* < 0.001). Pharyngeal NG was found in 9/33 (27.3%) female cases. Decreased susceptibility to ceftriaxone (MIC ≥ 0.03 mg/L) occurred in 2.5% NG isolates, none heterosexually acquired. All were azithromycin susceptible. A significant trend of increasing prevalence of heterosexual gonorrhoea in an urban Australian STD clinic setting is reported. We advocate maintenance of NG screening in women, including pharyngeal screening in all women with partner change who report fellatio, as pharyngeal NG may be an important reservoir for heterosexual transmission. Outreach to CSW should be enhanced.

## 1. Introduction

Latest surveillance indicates rising rates of *Neisseria gonorrhoea* (NG) in New South Wales, Australia [1]. The risk of HIV transmission is significantly enhanced by coinfection with NG [2, 3], and so the control of NG particularly in light of increasing minimum inhibitory concentration (MIC) values to ceftriaxone is a major public health concern [4, 5]. The predominance of gonorrhoea amongst Australian urban men who have sex with men (MSM) is well documented [1, 6] but heterosexual gonorrhoea in urban settings is less well characterised. NG is a notifiable disease in Australia but data is only collected by age, sex, and region of diagnosis and so heterosexual trends are poorly defined. Trends of increasing prevalence of heterosexually acquired NG and acquisition from fellatio and commercial sex worker (CSW) contact were noted in our suburban STD services in 2009, prompting this investigation specifically aimed at examining sexual behavioral aspects of heterosexual NG acquisition.

## 2. Methods

A case series was conducted from patient records at two STD services in South Eastern Sydney over a 5-year period, January 1, 2008 to December 31, 2012. Data was collected prospectively from late 2009 when the study started but retrospectively prior to this. These clinics operate in a culturally diverse suburban environment and offer free services by triage to high risk patients defined by “priority populations” specified in the 2010–2013 NSW Sexually Transmitted Infection (STI) strategy [7] (i.e., MSM, youth, CSW, multipartnered heterosexuals, intravenous drug users, HIV positive, indigenous) and other symptomatic patients, contacts of STDs, or those referred by General Practitioners (GPs). 

NG cases were identified from the clinic database. All NG cases were included whether detected by routine screening or testing in symptomatic patients. Heterosexual acquisition was defined as sexual activity not involving any same sex contact in the preceding 12 months. Heterosexual patient numbers were derived from total client numbers, minus MSM and women who have sex with women (WSW). Likely acquisition source and activity was identified from detailed sexual histories, which routinely seek information on the nature and timing of recent sexual contacts including number and sex of consorts, type of sexual contact (oral, vaginal, anal, insertive/receptive), and condom use for each activity. In deciding the likely transmission mode and source of NG, we took into account onset of symptoms and NG disease incubation time.

Receipt of oral sex (fellatio) was considered the likely route of NG infection when this activity occurred in isolation without a condom or if this occurred concurrently with vaginal or anal sex where a condom was used for the latter activities but not for oral sex. A female commercial sex worker was defined as a woman who stated she was currently engaged in sex work. Local contact was defined as sexual contact with a person in Australia.

Clinic policy states that all symptomatic patients are tested for NG in the relevant anatomical site. MSM are screened for NG in the rectum, urine, and throat, CSW are screened in the throat and cervix or urine, heterosexual men in the urine, and heterosexual women in the cervix or urine. Cases diagnosed by PCR are also cultured where possible, in order to ascertain antimicrobial susceptibility data. NG was treated during the study with ceftriaxone 250 mg IMI, and this was increased to 500 mg IMI from early 2010 in keeping with local recommendations. Cefixime is not available in Australia.

NG was cultured on selective media of lysed horse blood agar containing vancomycin, colistin, nystatin, and trimethoprim (VCNT) inhibitors. Antimicrobial susceptibility testing was performed prospectively at the Neisseria Reference Laboratory, Randwick, Sydney, a WHO Collaborating Centre for STD, using published methodology [8]. Decreased susceptibility of NG to ceftriaxone in extragenital sites was reported when the MIC value was ≥0.03 mg/L and ≥0.06 mg/L in genital sites [8]. All samples positive for NG using Nucleic Acid Amplification Techniques (NAAT) (Roche Amplicor PCR from study commencement to June 2011 and then from July 2011 by Roche Cobas 4800) were confirmed by supplementary assays targeting porA and opa genes as required by the National Testing Guidelines [9].

Proportions were compared using Chi-Square tests and trends identified using a Mantel-Haenszel Chi-Square test with SAS (version 9.2; SAS Institute Inc., Cary, NC, USA). 

Ethics approval was granted by the South Eastern Illawarra Area Health Service Ethics Committee.

## 3. Results

During the study 6164 patients were seen of which 5118 (83.0%) were classified as heterosexual and 1046 (17.0%) MSM or WSW, with approximately equal numbers of male (53.1%) and female (46.9%) heterosexual patients (Table 1). There were 167 cases of NG, 102 (61.1%) heterosexually acquired (overall prevalence 2.0%) and 65 (38.9%) MSM acquired (overall prevalence 6.9%). Over the 5-year period there was a significant trend of increasing NG prevalence in heterosexuals rising from 1.1% (95% CI 0.6–2.1) in 2008 to 3.0% (95% CI 2.0–4.0) in 2012 (*P* = 0.027).

Of heterosexual cases, 69 were males and 33 were females (M : F ratio 2.1 : 1). 67/69 (97.1%) of heterosexual males and 25/33 (75.8%) of females had genital symptoms. 

Receipt of unprotected fellatio was the likely source of acquisition for 21/69 (30.4%) of heterosexual males (11/21 CSW related and 10/21 non-CSW related). Commercial sex work (CSW) contact was the probable NG source for 28/69 (40.6%) of heterosexual males (18 CSW contacts local, and 10 whilst overseas). Only 7/33 (21.2%) of female cases reported current CSW. Importantly, NG prevalence during the study overall in CSW (2.2%) was not significantly higher than in non-CSW (1.2%) (*P* = 0.15), but in 2012 there was a significant increase in NG prevalence in CSW seen (8.6%) compared to non-CSW (1.6%), *P* < 0.001.

Of female cases, 31/33 (93.9%) reported unprotected vaginal sex. Pharyngeal NG was found in 9/33 (27.3%) women, 5 of these CSW. NG was acquired locally in 24/33 (72.7%) of females and 47/69 (68.1%) of heterosexual males. 

137/167 (82%) of NG cases were diagnosed by positive culture and 30/167 (18%) by positive PCR alone. Antimicrobial susceptibility data was available in 122/137 (89%) of NG cases diagnosed by culture. Decreased susceptibility to ceftriaxone was reported in 3/122 (2.5%), two pharyngeal and one rectal isolate all MSM related isolates, none heterosexually acquired. Of the 122 isolates with antibiotic susceptibility data, 55/122 (45.1%) were MSM related and 67/122 (54.9%) were heterosexually related. All NG isolates were azithromycin susceptible.

## 4. Discussion

This study found an increasing prevalence of heterosexual gonorrhoea in an urban Australian setting from 2008 to 2012, a trend which may be contributing significantly to rising NG notifications in Australia. At our services heterosexual acquisition accounted for 61.0% (102/167) of NG cases. The male : female ratio in heterosexual cases of 2.1 : 1 is comparable to the overall national Australian surveillance ratio of 2 : 1 [10]. The overall study male : female ratio including the MSM cases was 4.0 : 1, which is in marked contrast to our South East Sydney local health district reported ratio of 8 : 1 where NG detection predominates in the large MSM population [1, 10]. This reflects the lower proportion of MSM attendances at our clinics (25.8%) compared to inner city services. The persistence of the 2 : 1 male : female case ratio in heterosexuals is interesting. Factors contributing to this male predominance in heterosexual NG case detection might include increased likelihood of symptomatic disease and therefore detection in males (and more asymptomatic disease in females), lack of pharyngeal screening in females, suboptimal screening of CSW and high risk females, and possibly some misclassification of “heterosexual” acquisition. Enhanced surveillance of NG might help to clarify this enigma.

Receipt of unprotected fellatio was the likely sexual activity resulting in NG acquisition for 30% of heterosexual men. Additionally the number of heterosexual male NG cases due to fellatio may be underestimated in this study if receipt of unprotected oral sex occurred in conjunction with unprotected vaginal or anal sex. The pharyngeal NG reservoir in MSM is well recognized [11, 12], but this reservoir could also be important in all women with partner change who practice fellatio, not just CSW. This theory is supported by the high transmission rate in heterosexual men receiving fellatio and noting that equal numbers of fellatio-related transmissions occurred in men reporting contact with CSW and non-CSW females. Oral sex is frequent amongst heterosexuals and is typically unprotected. This combined with lack of awareness of the associated STD transmission risk, common perception that oral sex is not sex [13] and infrequent pharyngeal screening in women, may facilitate heterosexual NG transmission via this route. We isolated pharyngeal NG from 27% of female cases (by culture), but this is likely to be an underestimate due to clinic policy at the time of only undertaking pharyngeal screening in CSW and MSM. Increased uptake of more sensitive NAAT testing in the pharynx [14] is also likely to improve female NG detection. Our findings in both men and women suggest that an NG pharyngeal reservoir in women may be a common source of NG infection for heterosexual males. A recent UK study [15] also suggested that the pharynx may be an important NG reservoir in heterosexual women with a similar finding of 30% of female NG cases being pharyngeal. We found that NG infections in heterosexual men were almost always associated with genital symptoms (97.1%) but women less commonly so (75.8%). Hence, asymptomatic screening in women may be particularly important. Accordingly, our clinic guideline has changed to recommend the maintenance of NG screening in heterosexual women with additional pharyngeal NG screening in those women reporting partner change and fellatio. As female cases generally reported unprotected vaginal sex (93.9%), condom use is also reiterated. 

CSW in Australia have low rates of STDs reflecting good condom use [16]. However, a recent study of CSWs providing fellatio in Sydney [17] found that Cantonese speaking women were significantly less likely to use condoms for this service than Thai-speaking and English-speaking CSW. Additionally, women who do not identify as being CSW (e.g., working in massage) may be less likely to engage in safe sex, including safe oral sex [17]. Our study found that 40% of heterosexual males reported CSW contact but only 21% of female cases were CSW, an inconsistency which could reflect suboptimal testing rates and outreach to CSW in our population. Importantly NG prevalence in female CSW overall was no different from that in female non-CSW, except for the significant rise noted in 2012. Of concern, however, was the finding that 2/3 of the NG infections related to CSW contact occurred locally in Sydney, the rest acquired from overseas contacts. This would suggest a need to enhance local educational and testing services available to women engaged in CSW. 

Three quarters of all infections were acquired from local contact, reflecting the increasing local heterosexual NG prevalence noted in this study and from local surveillance [1]. Numbers of NG cases rose in 2012 which is also in keeping with the rise in local NG prevalence.

Antimicrobial susceptibility data was available for 89% of cultured isolates. Decreased susceptibility to ceftriaxone occurred in 3/122 (2.5%) isolates, all MSM related cases from extragenital sites. No decreased susceptibility was noted in heterosexually acquired isolates but numbers are too small to speculate on the significance of any difference in antibiotic susceptibilities between these populations at this time, but this should be the subject of ongoing monitoring and surveillance to inform treatment recommendations in these different populations. The finding of 3 isolates with decreased susceptibility to ceftriaxone is consistent with the right shift in MIC values to ceftriaxone reported locally [4] and globally [5, 18] and cause for concern for disease control in the absence of viable treatment alternatives if resistance to ceftriaxone develops. All isolates were sensitive to azithromycin. We recommend that cases positive by PCR should also be cultured where possible for purposes of monitoring NG isolate susceptibility. The widespread supplanting of culture methods with PCR has the advantages of greater sensitivity and no fuss specimen transport, but at the cost of antimicrobial surveillance. STD services are best placed to maintain this NG antimicrobial resistance surveillance role.

This study is limited by small numbers, reliance on patient sexual histories, and its partially retrospective nature in two triaged STD clinic populations within the same local health district. Inevitably some definitions particularly relating to transactional sex can become blurred and sexual histories are reliant on patient recall and propensity to disclose the exact nature of the contact. Some cases were unwilling or unable to identify the likely source of the infection. Clinical judgment was applied to determine the likely source of infection based on disease incubation, onset of symptoms, and detailed recent sexual history. Triage processes were unchanged over the study period and priority population groups remained relatively stable. Importantly antibiotic resistance testing was prospective and performed in a reference laboratory, a WHO Collaborating Centre for STD.

## 5. Conclusion

A significant trend of increasing prevalence of heterosexual gonorrhoea in an urban Australian STD clinic setting is reported. This study suggests that the pharynx may be an important reservoir for heterosexual NG transmission, and we advocate maintenance of NG screening in women, particularly inclusion of pharyngeal screening in women with partner change who practice fellatio. Case detection, enhanced surveillance, and health promotion are pivotal to NG control. Health promotion efforts should include messages concerning STD transmission risks associated with oral sex in heterosexuals, and we recommend enhanced CSW engagement with education and STD testing opportunities.

## Figures and Tables

**Table 1:** Summary of heterosexual patients and NG cases 2008–2012.

	2008	2009	2010	2011	2012	Overall
No. hetero patients (male and female)	875	1009	1102	1041	1091	5118
No. hetero male patients	473	529	577	539	599	2717
No. hetero female patients	402	480	525	502	492	2401
Total NG cases	18	35	27	33	54	167
Total hetero NG	10	26	13	20	33	102
Hetero male NG	5	19	11	13	21	69
Hetero female NG	5	7	2	7	12	33
Prevalence of NG in heterosexuals (%)∗	1.14	2.58	1.18	1.92	3.02	2.0
No. female CSW	50	63	76	75	58	322
Mean age (yrs) hetero males with NG∗∗	32.4	33.1	38.2	44.3	37.4	37.3
Mean age (yrs) hetero female with NG∗∗∗	32.4	39.0	44.0	25.7	32.8	33.2

^*^A significant trend of increasing NG prevalence in heterosexuals was noted over the 5 year period (*P* = 0.027).

∗∗No significant age trend in heterosexual male NG cases (*P* = 0.122).

∗∗∗No significant age trend in heterosexual female cases (*P* = 0.387).
